# An Epigenetic Feedback Regulatory Loop Involving MicroRNA-195 and MBD1 Governs Neural Stem Cell Differentiation

**DOI:** 10.1371/journal.pone.0051436

**Published:** 2013-01-17

**Authors:** Changmei Liu, Zhao-Qian Teng, Andrea L. McQuate, Emily M. Jobe, Christa C. Christ, Sergei J. von Hoyningen-Huene, Marie D. Reyes, Eric D. Polich, Yina Xing, Yue Li, Weixiang Guo, Xinyu Zhao

**Affiliations:** 1 Cellular and Molecular Biology Graduate Program, University of Wisconsin-Madison, Madison, Wisconsin, United States of America; 2 Department of Neurosciences, University of New Mexico School of Medicine, Albuquerque, New Mexico, United States of America; 3 Waisman Center, University of Wisconsin-Madison, Madison, Wisconsin, United States of America; 4 Department of Neuroscience, University of Wisconsin-Madison, Madison, Wisconsin, United States of America; University of Massachusetts, United States of America

## Abstract

**Background:**

Epigenetic mechanisms, including DNA methylation, histone modification, and microRNAs, play pivotal roles in stem cell biology. Methyl-CpG binding protein 1 (MBD1), an important epigenetic regulator of adult neurogenesis, controls the proliferation and differentiation of adult neural stem/progenitor cells (aNSCs). We recently demonstrated that MBD1 deficiency in aNSCs leads to altered expression of several noncoding microRNAs (miRNAs).

**Methodology/Principal Findings:**

Here we show that one of these miRNAs, miR-195, and MBD1 form a negative feedback loop. While MBD1 directly represses the expression of miR-195 in aNSCs, high levels of miR-195 in turn repress the expression of MBD1. Both gain-of-function and loss-of-function investigations show that alterations of the MBD1–miR-195 feedback loop tip the balance between aNSC proliferation and differentiation.

**Conclusions/Significance:**

Therefore the regulatory loop formed by MBD1 and miR-195 is an important component of the epigenetic network that controls aNSC fate.

## Introduction

Upon completion of initial embryonic development, neurogenesis persists in restricted brain regions, such as the dentate gyrus (DG) of the hippocampus. The cellular basis of postnatal and adult neurogenesis is neural stem/progenitor cells (aNSCs) residing in these adult germinal zones. The aNSCs can self-renew and are multipotent, capabilities that are tightly controlled by intricate molecular networks [1], [2], [3]. Although the functional properties of aNSCs have been studied extensively, we do not yet fully understand the detailed molecular mechanisms controlling the maintenance and fate specification of aNSCs. Deciphering these regulatory mechanisms is critical for understanding adult brain plasticity, as well as for developing cell-based therapies for brain diseases.

Recent research has shown that epigenetic regulation plays significant roles in the modulation of stem cell proliferation and differentiation [4], [5], [6]. One of the most exciting findings in the past few years is that developmental processes are regulated by the crosstalk between epigenetic modulators, including post-translational modifications of nucleosomal histones, changes in histone variants, chromatin remodeling enzymes, DNA methylation, and microRNAs [4], [5]. Methylated-CpG binding proteins (MBDs), including MBD1 and MeCP2, regulate gene expression by recognizing genomic DNA methylation [7]. Despite the fact that MBD1 is expressed ubiquitously, MBD1 deficiency in mice results largely in brain-associated phenotypes, including impaired adult neurogenesis, defective hippocampus-dependent learning, and susceptibility to depression [8], [9]. Not surprisingly, functional deficiencies of MeCP2 and MBD1 are associated with human neurodevelopmental disorders [10], [11], [12]. We have shown that MBD1 deficiency selectively decreases the ability of aNSCs to differentiate, in part through its epigenetic repression of the stem cell mitogen FGF-2 [4]. However, the downstream effectors mediating MBD1’s regulation of neurogenesis remain for the most part a mystery [4], [9], [13].

MicroRNAs (miRNAs) are a recently discovered large family of 20–22–nucleotide non-coding RNAs that are involved in numerous cellular processes, including stem cell proliferation and differentiation [6], [14], [15]. Although the precise mechanism is still being worked out, extensive experimental evidence shows that miRNAs regulate gene expression by targeting RNA-induced silencing complex to specific messenger RNAs. Specific miRNAs are known to modulate the functions of many types of stem cells, including aNSCs [16], [17], [18], [19], and certain groups of miRNAs are brain-specific or enriched in the brain [20]. Among these miRNAs, miR-195 exhibits distinct developmental and lamina-specific expression in human prefrontal cortex [21] and is a core regulator in modulating the expression of schizophrenia-related genes [22]. We previously showed that miR-195 is one of the miRNAs with increased expression levels in MBD1-deficient aNSCs, but the function of miR-195 in aNSC proliferation and differentiation is unclear. Moreover, we do not know how the expression of miR-195 is controlled in aNSCs.

Here we provide evidence that MBD1 directly represses the expression of miR-195 in aNSCs derived from the DG of the adult hippocampus, and miR-195 in turn represses MBD1 expression through the seed sequence within the 3′ untranslated region (UTR) of *Mbd1* mRNA. Whereas MBD1 promotes aNSC differentiation, miR-195 represses aNSC differentiation. Alterations of the MBD1–miR-195 feedback loop via changes in the levels of either miR-195 or MBD1 tip the balance between aNSC proliferation and differentiation in vitro and in vivo. Hence, MBD1 and miR-195 form an epigenetic feedback loop that regulates aNSC proliferation and differentiation.

## Results

### MBD1 Regulates the Expression of miR-195 in aNSCs

**Figure 1:**
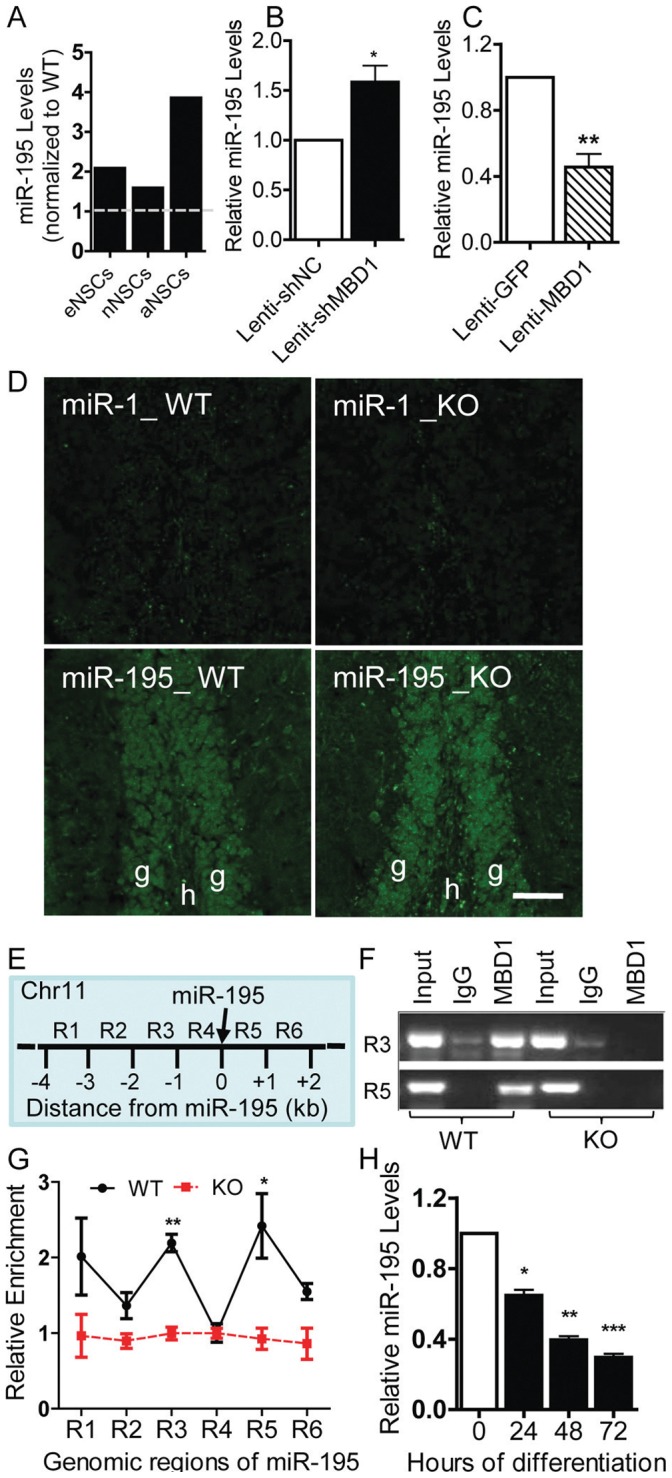
MBD1 regulates miR-195 expression in aNSCs. (A): The expression of miR-195 was increased in *Mbd1 KO* proliferating NSCs derived from embryonic (E14.5) telencephalon cells (eNSCs), neonate (P0) forebrain cells (nNSCs), and adult DG cells (aNSCs). Real-time PCR analyses were performed using independently isolated cells; average values from two experiments were presented for eNSCs and nNSCs, and from one experiment for aNSCs. (B): Acute knockdown of MBD1 resulted in increased miR-195 expression in aNSCs (n = 5, p<0.5). (C) Overexpression of MBD1 in aNSCs led to reduced miR-195 expression (n = 5, p<0.5). (D) Fluorescence in situ hybridization (FISH) analyses showing expression of miR-195 in the dentate gyrus (the bottom two panels) of WT and *Mbd1 KO* mice (green: miRNA probe signal). (E) Schematic drawing of the 5-kilobase (kb) regions proximal to the miR-195 gene on chromosome 11 that were assayed in ChIP experiments. An increase in staining intensity for miR-195 was seen in *Mbd1 KO* brains. miR-1 probe was used as a negative control for FISH. Scale bars = 40 µm. g, granule cells of the dentate gyrus. h, hilar region of the dentate gyrus. (F) Chromatin immunoprecipitation (ChIP) assay demonstrating that MBD1 bound to two genomic regions, R3 and R5, corresponding to 2 kb upstream and 1 kb downstream, respectively, of miR-195 genomic, but not in KO brains. (G) ChIP assay followed by real time PCR analysis using 6 sets of primers covering the −4 kb to +2 kb of miR-195 genomic region demonstrates the enrichment of MBD1 protein at genomic sequence 2 kb upstream and 1 kb downstream of the miR-195 locus in WT aNSCs. Quantities were calculated from an input DNA-generated standard curve. Relative enrichment of MBD1 in either WT or KO aNSCs was calculated relative to IgG-only nonspecific control in the same cells (n = 3). Two-way ANOVA, Bonferroni post-test was used for data analyses. For both E and F, IgG-ChIP in WT aNSCs and MBD1-ChIP antibody in *Mbd1 KO* aNSCs were used as negative controls. (H) The expression levels of miR-195 decreased upon aNSC differentiation. Data are presented as mean ± SEM; *, p<0.05, **, p<0.01, One-sample t-test was used for data analyses.

We previously identified a subset of miRNAs that exhibit altered expression in MBD1-deficient aNSCs and showed that miR-184 is regulated by MBD1 [18]. Further analysis using miRNA real-time PCR assays confirmed that the miR-195 level was indeed increased in NSCs isolated from the DG of *Mbd1 KO* mice (Fig. 1*A*), consistent with previous miRNA array data [18]. To determine whether MBD1 directly regulates the expression of miR-195 in aNSCs, we acutely manipulated MBD1 expression in adult DG-derived aNSCs (DG-aNSCs, which will be referred to as aNSCs hereafter) using lentivirus expressing either MBD1 coding sequence or a small inhibitory RNA against MBD1 (Lenti-shMBD1). As expected, we found that acute knockdown of MBD1 in aNSCs led to increased miR-195 expression levels (Fig. 1*B*), whereas overexpression of MBD1 resulted in decreased miR-195 expression levels (Fig. 1*C*). We then confirmed that miR-195 was expressed in the adult DG (Fig. 1*D*) in a pattern similar to that of MBD1 [9], [18], and we did see a consistent increase in miR-195 signal in *Mbd1 KO* mouse brains compared to wild-type (WT) brains.

To determine whether MBD1 interacts directly with genomic regions proximal to miRNA-195, we conducted chromatin immunoprecipitation (ChIP) using an anti-MBD1 antibody and examined the association of MBD1 with DNA sequences spanning −4 kb upstream to +2 kb downstream relative to the position of pre-miR-195 (Fig. 1*E*). We found that 2 genomic regions, R3 (−1 kb upstream) and R5 (1 kb downstream), could be specifically amplified from MBD1-ChIP DNA (Fig. 1*F*). We then performed a quantitative evaluation of MBD1 binding to the miR-195 genomic region by using ChIP coupled with real-time quantitative PCR. We found that MBD1 was enriched 2.6-fold at the R3 region and 1.8-fold at the R5 region in WT aNSCs compared with the two negative controls, IgG-IP in WT cells and MBD1-IP in *Mbd1 KO* aNSCs (Fig. 1*G*).

In addition, we found that the expression levels of miR-195 were decreased during aNSC neuronal differentiation (Fig. 1*H*). Since MBD1 expression increases during aNSC neuronal differentiation [18], this observation lends further support to a negative regulatory relationship between miR-195 and MBD1. Taken together, these data argue for the idea that MBD1 represses miR-195 expression in DG-aNSCs by directly binding to the genomic regions proximal to pre-miR-195.

### miR-195 Regulates the Differentiation of aNSCs in vitro

**Figure 2:**
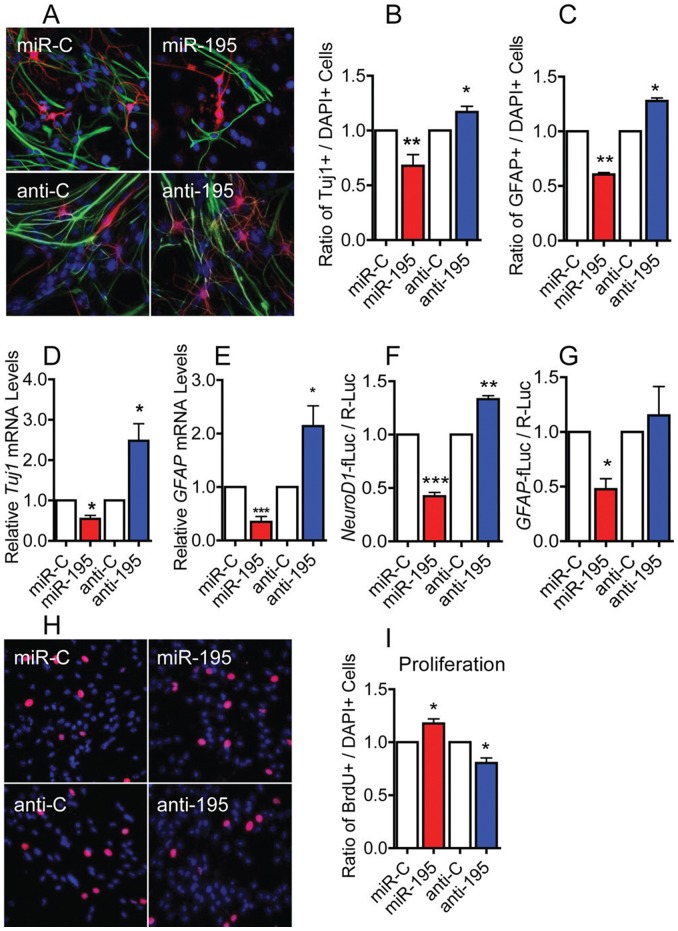
miR-195 modulates the differentiation and proliferation of adult NSCs in vitro. (A) DG-aNSCs were transfected with either synthetic miR-195 mimics or control miRNA mimics (miR-C), and aNSC differentiation was analyzed using neuronal lineage marker Tuj1 (red) and astrocyte lineage marker GFAP (green). DAPI, blue. (B, C) DG-aNSCs transfected with miR-195 mimics led toreduced neuronal (B, n = 4, p<0.5) and astrocyte (C n = 3, p<0.5)) differentiation, compared to cells transfected with miR-C. On the other hand, DG-aNSCs transfected with a specific inhibitor to miR-195 (anti-195) led to increased neuronal (B) and astrocyte (C) differentiation, compared to cells transfected with Anti-C. (D, E), aNSCs transfected with miR-195 exhibited reduced neuronal (D) and astrocyte (E) differentiation, whereas aNSCs transfected with anti-195 had increased neuronal (D) and astrocyte (E) differentiation as assessed via real-time PCR analyses for mRNA levels of neuronal gene *Tuj1* (D) and astrocyte gene *GFAP* (E). (n = 6 to 8, P<0.05). (F, G), aNSCs transfected with miR-195 exhibited reduced neuronal (F) and astrocyte (G) differentiation, whereas aNSCs transfected with anti-195 had increased neuronal (F) and astrocyte (G) differentiation as assessed by the promoter activities of NeuroD1 (F, for neurons, n = 3 for miR; n = 5 for anti-miR, p<0.01) and GFAP (G, for astrocytes n = 4, p<0.05 for miR). (H) DG-aNSCs were transfected with either miR-195 mimics or miR-C, and aNSC proliferation was analyzed using BrdU labeling (red = DAPI, blue. (I). Quantitative analysis indicates that miR-195-transfected aNSCs had increased BrdU incorporation, compared to miR-C–transfected cells (n = 8 for miR; n = 6 for anti-miR, p<0.05). On the other hand, anti-195-transfected aNSCs displayed reduced BrdU incorporation compared to anti-Control transfected aNSCs (n = 9; p<0.05). Data are presented as mean ± SEM; *, p<0.05, **, p<0.01, One-sample t-test was used for data analyses.

To explore the role miR-195 plays in aNSC differentiation, we transfected aNSCs with either a synthetic miR-195 RNA mimic (miR-195) or a miR-195 inhibitor (anti-195) and subjected the transfected aNSCs to differentiation treatment (Fig. 2*A*; Fig. S1 in File S1). Our data revealed that aNSCs transfected with miR-195 differentiated into fewer neurons and astrocytes, whereas aNSCs transfected with anti-195 exhibited increased neuronal and astrocyte differentiation as assessed by the proportions of cells expressing neuronal and astrocyte lineage markers (Fig. 2*B and C*). These results were validated by quantitative analysis of the mRNA levels of the neuronal marker Tuj1 (Fig. 2D) and the glial marker GFAP (Fig. 2*E*). To further validate the above results, we assessed neuronal and glial promoter activities using luciferase as reporters [18], [23], [24], and the results showed that overexpression of miR-195 led to reduced activities of *NeuroD1* and *GFAP* promoters, whereas blocking the function of endogenous miR-195 using anti-195 resulted in enhanced *NeuroD1* promoter activity (Fig. 2*F and G*). Therefore, high levels of miR-195 inhibited the neuronal and astrocyte differentiation of aNSCs.

To determine whether miR-195 affected aNSC proliferation, we used BrdU pulse-labeling (Fig. 2*H*, Fig. S2 in File S1). The aNSCs transfected with miR-195 exhibited 13.2% more BrdU incorporation than control miR-C-transfected cells (Fig. 2*I*). On the other hand, aNSCs transfected with anti-195 had 12.1% less BrdU incorporation than aNSCs transfected with anti-C (Fig. 2*I*). Thus, high levels of miR-195 enhance proliferation and repress differentiation of aNSCs.

### miR-195 Regulates the Differentiation of aNSCs in vivo

**Figure 3:**
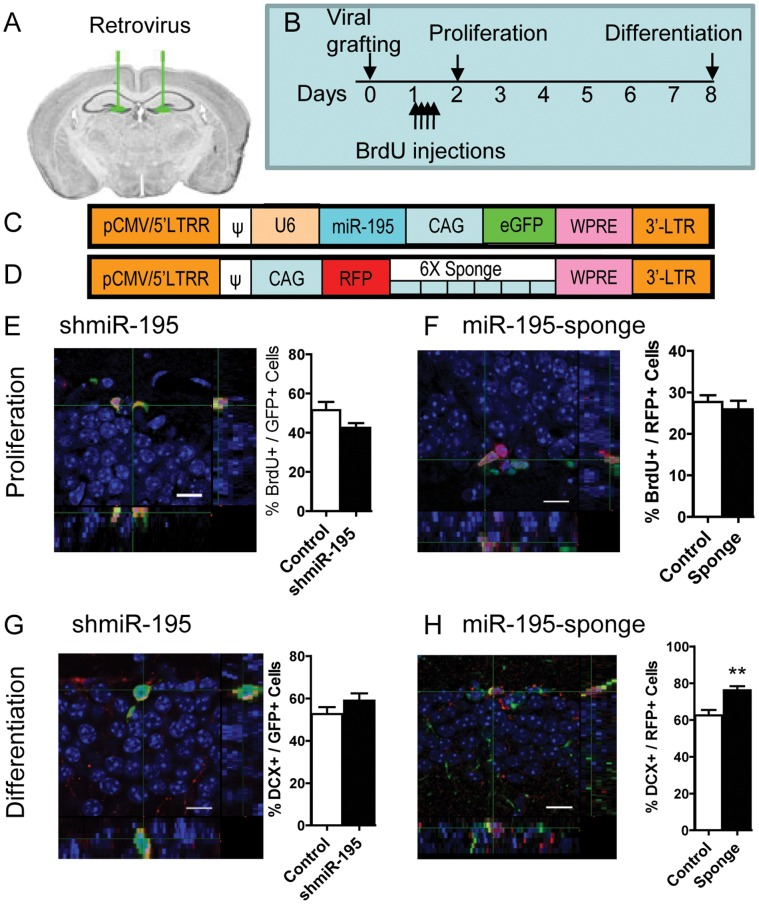
miR-195 modulates differentiation of adult NSCs in vivo. (A, B) Experimental scheme for assessing aNSC proliferation and early stage differentiation in adult mice after retrovirus grafting. The experimental and control viruses were grafted on either side of the same animals. (C). Schematic drawing of the retroviral vector used for expressing miR-195 or sh-NC. Sh-miR-195 was expressed as a short hairpin driven by U6 RNA polymerase III promoter, while GFP was expressed driven by a chicken actin (CAG) promoter. (D). Schematic drawing of the retroviral vector used for expressing miR-195-sponge. The 6 tandem repeats of miR-195-sponge were inserted in the 3′ region of RFP, therefore the expression of both RFP and miR-195-sponge were driven by the CAG promoter. (E-H) Left panels show representative 3D Z-stack images of BrdU^+^GFP^+^ (E), BrdU^+^RFP^+^ (F), DCX^+^GFP^+^ (G), and DCX^+^RFP^+^ (H) cells, used for quantification. Right panels shows quantification of retrovirus-infected cells that have incorporated BrdU (E, F) or differentiated into neurons (G, H). (E, F) Compared with control retrovirus-infected cells, neither shmiR-195 retrovirus (E, control n = 6, shmiR-195 n = 4, p = 0.14), nor miR-195-sponge virus (F, control n = 4, sponge n = 4, p = 0.52), had significant effect on BrdU incorporation of infected aNSCs. (G–H) although shmiR-195 retrovirus did not alter neuronal differentiation (G, control n = 6, shmiR-195 n = 6, p = 0.22), a higher percentage of miR-195-sponge retrovirus-infected cells was positive for DCX (H, control n = 4, sponge n = 5, p = 0.007). Data are presented as mean ± SEM; statistics were done using student t-test **, p<0.01.

To assess the functions of miR-195 in aNSCs in vivo, we decided to use a retrovirus-based single cell genetics approach (Fig. 3*A and B*) [2], [18], [19], [25], [26], [27], [28], [29]. We constructed a retroviral vector that expressed both short hairpin pre-miR-195 (shmiR-195) driven by a U6 promoter and GFP by a chicken actin (CAG) promoter (Fig. 3*C*) for our gain-of-function assay. We also created a miR-195-sponge [30] to inhibit endogenous miR-195 (Fig. 3*D*) for our loss-of-function assay. We confirmed that shmiR–195 virus-infected aNSCs exhibited increased miR-195 levels (Fig. S3A in File S1) and reduced neuronal differentiation (Fig. S3B in File S1), whereas miR-195-sponge virus–infected aNSCs enhanced neuronal differentiation (Fig. S3B in File S1) compared to corresponding controls. The negative controls, including retrovirus-shNC, retrovirus-GFP (CAG-GFP), and retrovirus-mRFP (CAG-RFP), had no effect on aNSC differentiation [4], [18], [24], [26].

To determine the function of miR-195 in aNSC differentiation in vivo, retrovirus expressing either shmiR-195 (also GFP), miR-195-sponge (also RFP) or control retroviruses (shNC as control for retro-miR-195, or CAG-RFP for retro-miR-195-sponge) was stereotaxically grafted into the DG of adult animals (Fig. 3*A*). Mice also received BrdU injections immediately after the surgery to label dividing cells. At 12 hours post-viral injection, a group of animals were sacrificed to determine cell proliferation. At one week post-viral injection, we analyzed differentiation of viral-infected cells using an early neuronal marker, doublecortin (DCX) (Fig. S4 in File S1). Using Z-stack images of confocal microscopy at 1-µm resolution, we quantified the percentage of retrovirus-labeled (GFP+ or RFP+) cells that expressed either DCX or incorporated BrdU. Compared with control retrovirus-infected cells, neither shmiR-195 retrovirus (Fig. 3*E*, BrdU+GFP+/GFP+), nor miR-195-sponge virus (Fig. 3*F*, BrdU+RFP+/RFP+) had significant effect on aNSCs BrdU incorporation. On the other hand, although shmiR-195 retrovirus did not alter neuronal differentiation (Fig. 3*G*, DCX+GFP+/GFP+), a higher percentage of miR-195-sponge retrovirus-infected cells were DCX-positive (Fig. 3*H*, DCX+RFP+/RFP+). Therefore, inhibition of endogenous miR-195 levels promotes neuronal differentiation of aNSCs. These results suggest that the level of endogenous miR-195 in aNSCs is an important regulatory mechanism for neuronal differentiation.

### MBD1 is a Target of miR-195 in aNSCs

**Figure 4:**
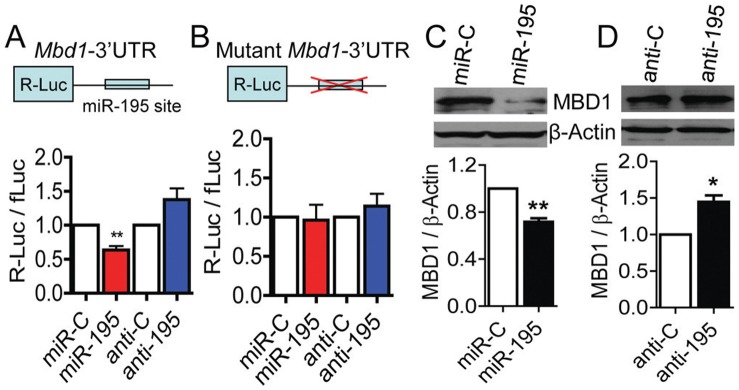
MBD1 is a functional target of miR-195. (A) The *Mbd1*-3′-UTR–dependent expression of Renilla luciferase reporter gene (R-Luc) was suppressed by miR-195 (n = 5, p<0.01) and enhanced by the inhibitor of miR-195 (anti-195) in aNSCs (n = 5, p = 0.087) compared with corresponding controls. (B) The mutation of the miR-195 target site in the *Mbd1* 3′ UTR abolished the repression by miR-195 and the enhancement by anti-195, meaning miR-195–mediated suppression of luciferase-*Mbd1*-3′-UTR was dependent on miR-195 target (n = 6, p>0.05). (C, D) Representative Western blot images (top panels) and quantification of independent Western blots (n = 4) showed that overexpression of miR-195 in WT aNSCs led to reduced endogenous MBD1 protein expression (C), whereas inhibition of miR-195 by anti-195 led to enhanced MBD1 protein expression in *Mbd1 KO* aNSCs (D). Data are presented as mean ± SEM; statistics were done using Student’s t-test*, p<0.05, **, p<0.01.

miRNAs are known to function at least in part by repressing protein translation of their mRNA targets [15], [31], [32]. We therefore searched for potential mRNA targets of miR-195 by cross-referencing two widely used miRNA target prediction programs, TargetScan and miRanda. Several miR-195 targets were predicted by both software programs, including *Mbd1*, *Bdnf*, *Wee1*, *Mib1*, and *CyclinD1*, and we have validated that miR-195 can indeed repress Renilla luciferase (R-Luc) expression via the 3′ UTRs of some of these predicted targets (Table S1 in File S1). Surprisingly, we found that MBD1 was among the list of potential targets of miR-195 predicted by both programs, and the *Mbd1* 3′ UTR contained a classic miR-195 target seed sequence (Fig. S5 in File S1), suggesting that MBD1 and miR-195 might form an important regulatory loop in aNSC regulation. Indeed, high levels of miR-195 could repress the expression of R-Luc through the *Mbd1* 3′ UTR, whereas anti-195 enhanced R-Luc expression (Fig. 4*A*). In addition, retro-shmiR-195 could also repress the expression of R-Luc through the *Mbd1* 3′ UTR, whereas retro-miR-195-sponge also enhanced R-Luc expression (Fig. S6 in File S1), further supporting a regulatory role for miR-195 on MBD1 expression. We then mutated the miR-195 seed sequence within the *Mbd1* 3′ UTR (Fig. S5 in File S1). As predicted by our previous data, the mutation abolished both miR-195–mediated suppression and anti-195–mediated enhancement of R-Luc activities (Fig. 4*B*). These results strongly suggest that miR-195 directly represses MBD1 expression through the predicted target seed sequence located in the *Mbd1* 3′ UTR.

Next we determined the effect of miR-195 on endogenous MBD1 expression in aNSCs. We found that miR-195–transfected aNSCs showed a reduction in MBD1 protein levels (Fig. 4*C*), whereas anti-195 transfection rescued the levels of MBD1 expression in *Mbd1 KO* aNSCs (Fig. 4*D*). Taken together, these data argue that MBD1 is a direct target of miR-195, therefore MBD1 and miR-195 form a negative regulatory loop in aNSCs.

### MBD1 Rescues the Functional Phenotypes Resulting from Either miR-195 Overexpression or MBD1 Deficiency in aNSCs

**Figure 5:**
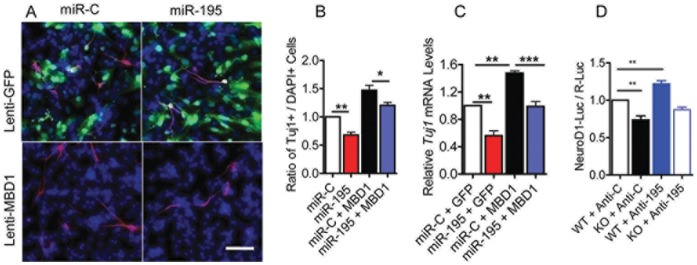
MBD1 rescues aNSC deficits associated with miR-195 overexpression. (A) Sample images showing DG-aNSCs infected with lentivirus expressing GFP as control (upper panel, GFP, green) or lentivirus expressing MBD1 (lower panel). The infected cells were then transfected with either miR-C (left panels) or miR-195 (right panels). The neuronal differentiation was assessed by using neuronal marker Tuj1 (red). DAPI, blue. (B) Overexpression of MBD1 rescued the neuronal differentiation deficits resulting from miR-195 overexpression as assessed by quantification of Tuj1+ cells over total (DAPI+) cells (Black bar v. blue bar, n = 4, p<0.05). (Same data for miR-C and miR-195 presented in Fig. 2B are used here for comparison purposes). (C) Overexpression of MBD1 rescued the neuronal differentiation deficits resulting from miR-195 overexpression as assessed by *Tuj1* mRNA levels (n = 6). The color of bars in C and D represents the same conditions as B. (D) Inhibition of endogenous miR-195 using a specific inhibitor (Anti-195) rescued the neuronal differentiation deficits resulting from MBD1 deficiency as assessed by NeuroD1 promoter activities (n = 6). Data are presented as mean ± SEM; statistics were done using one-sample t-test*, p<0.05, **, p<0.01.

We next used several methods to assess whether MBD1 could rescue the aNSC phenotypic changes that result from miR-195 overexpression. Consistent with our previous results, overexpression of miR-195 alone led to decreased neuronal differentiation (Fig. 5*A–C*, red bars), and overexpression of MBD1 alone resulted in increased neuronal differentiation (Fig. 5*B–C*, black bars; Fig. S7*A* in File S1). Expression of MBD1 rescued the neuronal differentiation deficits caused by miR-195 overexpression (Fig. 5*B–C*, blue bars; Fig. S7*A* in File S1). We went on to assess whether inhibition of miR-195 could rescue the phenotypes resulting from MBD1 deficiency. We found that transfection of a miR-195 inhibitor (anti-195) rescued the neuronal differentiation deficits of *Mbd1 KO* aNSCs (Fig. 5*D*). These data argue strongly that MBD1 is a functional target of miR-195 in aNSCs.

**Figure 6:**
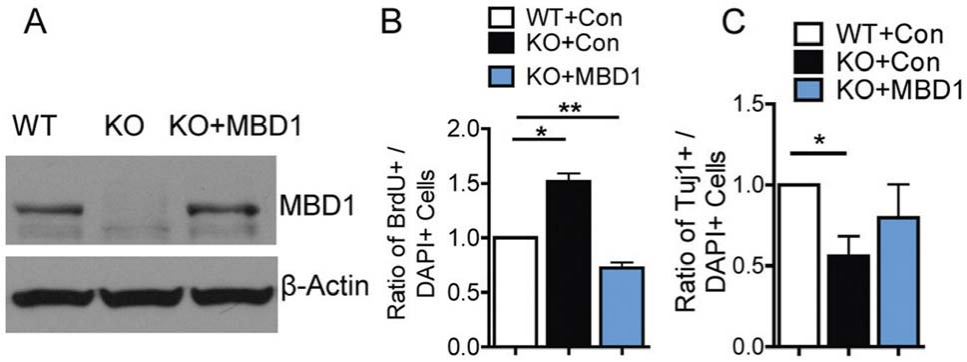
MBD1 rescues aNSC deficits associated with MBD1 deficiency. (A) Infection by lenti-MBD1 virus could restore MBD1 expression in *Mbd1 KO* aNSCs to levels similar to WT aNSCs. (B) Exogenous MBD1 rescued proliferation deficits exhibited by *Mbd1 KO* aNSCs (n = 4). (C) Exogenous MBD1 could rescue neuronal differentiation deficits exhibited by *Mbd1 KO* aNSCs as assessed by quantification of Tuj1-positive cells (C, n = 4, p<0.05). Data are presented as mean ± SEM; statistics were done using one-sample t-test*, p<0.05, **, p<0.01.

Finally, we investigated whether exogenous MBD1 expressed by a lentiviral vector (Fig. 6*A*) could rescue the phenotypic deficits exhibited by *Mbd1 KO* aNSCs; indeed, we found that expressed MBD1 suppressed proliferation (Fig. 6*B*) and enhanced neuronal differentiation (Fig. 6*C* *;* Fig. S7*B* in File S1) of *Mbd1 KO* aNSCs. Taken together, these results support our model in which MBD1 and miR-195 form a negative regulatory loop in aNSCs to control each other’s expression levels, and the delicate balance of this loop is important for governing the proliferation and differentiation of aNSCs.

## Discussion

In this study, we show that miR-195 and MBD1 form a negative feedback loop regulating the differentiation of aNSCs in the adult DG. We provide in vitro and in vivo evidence that alteration of the MBD1–miR-195 feedback loop can tip the balance between aNSC proliferation and differentiation, meaning this epigenetic regulatory loop is likely an important component of the epigenetic network that controls aNSC fate.

Recent publications from our own and other groups show that the crosstalk between miRNA and DNA methylation is probably a common mechanism regulating mammalian neurogenesis and development [18], [19], [33], [34], [35], [36], [37]. The present study is a clear example supporting this notion. Our previous studies have shown that MBD1 plays an important role in adult hippocampal neurogenesis [9], in part by repressing the expression of FGF-2 and miR-184 [4], [18]. MBD1 is expressed at higher levels in neurons and lower levels in aNSCs [4], [18]. In contrast, the level of the mature form of miR-195 decreases upon aNSC differentiation. The relative expression patterns between miR-195 and MBD1 resemble those seen between miR-9 and its target gene, TLX, and miR-124 and its target gene, REST [16], [38]. In these examples, miRNA target proteins are expressed at high levels in those cells where the miRNAs are found at low levels. Conversely, the expression of these mRNA targets is downregulated as their regulatory miRNAs accumulate. These inverse relationships support negative regulatory roles for these miRNAs and their mRNA targets. The significance of MBD1 binding to the R3 and R5 genomic regions of miR-195 remains a question. These two regions host a number of potential regulatory elements with interspersed CpGs, whereas the R4 region contains a classic CpG island. Since MBD1 does not bind to the R4 region at all, this indicates that binding to interspersed CpGs, either methylated or unmethylated, is important for MBD1 regulation of miR-195. Further analysis is underway to solve this question. In addition to miR-195, MBD1 likely also represses the expression of other miRNAs [18], and additional studies on these other miRNAs may further bolster this relationship.

Although several studies have demonstrated important functions for miR-195 in diverse biological systems (for example, miR-195 regulates the proliferation of cancer cell lines [39], [40], [41] and contributes to multidrug resistance in head and neck squamous cell carcinoma cell lines [42]), our study is the first to investigate the role of miR-195 in non-transformed primary neural progentitors. While Xu and colleagues [39] show that in human hepatocellular carcinoma cells, miR-195 targets Cyclin D1, CDK6, and E2F3 to suppress cell proliferation, we found that the overexpression of miR-195 promoted aNSC proliferation in vitro and had no effect on Cyclin D1 expression in aNSCs (Table S1 in File S1). Notably, these differences could be due to the different biological systems studied. This is particularly relevant when comparing transformed cancer cells and primary stem cells. The targets of miR-195 and the effects of miR-195 on cell proliferation may in fact be context-dependent, which underscores the importance of using primary cells to study normal biological pathways. In addition to MBD1, miR-195 may also exert its function through other effectors and pathways, which converge at the level of the genes directly controlling or associated with NSC fate, such as NeuroD1 and GFAP. Furthermore, we found that inhibiting miR-184, another MBD1-regulated miRNA, rescued aNSC neuronal differentiation deficit resulted from miR-195 overexpression (data not shown). Studies into additional targets of miR-195 and its interaction with other MBD1-regulated miRNAs are currently underway.

Our discovery of the MBD1–miR-195 regulatory loop and its implications in the control of aNSC fate offer exciting evidence for the role of a complex gene regulatory network in aNSC biology. The control of gene expression by autoregulatory feedback loops has long been known as a common regulatory mechanism that is particularly important during cell fate determination and development [43]. miRNAs are uniquely suited to participate in these feedback circuits, owing to their potential to repress mRNAs that encode factors involved in the regulation of the same miRNAs [43]. More and more evidence now points to miRNAs regulating their own transcription through negative or positive feedback loops with specific transcription factors. For example, miR-133b, a miRNA expressed specifically in midbrain dopaminergic neurons and deficient in the midbrain tissue of patients with Parkinson’s disease, is found to regulate the maturation and function of midbrain dopaminergic neurons within a negative feedback circuit that includes the paired-like homeodomain transcription factor PITX3 [44]. PITX3 stimulates transcription of miR-133b, which in turn suppresses PITX3 expression [43]. There are also negative feedback regulations between CyclinD1 and miR-17/20, between miR-200 and ZEB1, and between miR-214 and EZH2 [45], [46], [47]. In neural stem cells, miR-9 and TLX form feedback loops to control NSC proliferation and differentiation [16]. Here we found that the MBD1–miR-195 loop controls the differentiation of aNSCs. Thus, the regulatory feedback loop between miRNAs and transcription factors is likely to be an important common mechanism for regulating stem cell fate. Deciphering such a mechanism marks a step towards unraveling the regulatory network that underlies adult brain neurogenesis and gives us more clues about how to use neural stem cells as a potential therapy.

## Methods

### Isolation and Culture of Adult NSCs

All animal procedures were performed according to protocols approved by the University of New Mexico Animal Care and Use Committee. The *Mbd1* mutant mice used in this study were created by deleting exons 2–10 of the *Mbd1* genes [9] followed by crossing to C57BL6 background for >9 generations. Adult mouse brain-derived NSCs used in this study were isolated from the DG of 8- to 10-week-old male *MBD1* KO mice and wild-type (WT) controls. Isolation of embryonic NSCs (eNSCs) from E14.5 telencephalon and neonate NSCs (nNSCs) from P0 forebrain were performed as described [48]. The isolation of NSCs was performed according to published methods: for DG aNSCs [24], [29], [49]. After enzymatic digestion using MACS Neural Tissue Dissociation kit (Miltenyi Biotech, Germany), we added 5 ml of DMEM/F-12 containing 10% FBS (Sigma-Aldrich, #F 4135), 2 mM L-glutamine (GIBCO, #25030-081), and 1% Antibiotic-Antimycotic (GIBCO, #15240-062) into each sample to stop digestion. After filtering through a 70-µm cell strainer (BD Falcon, #252350, CA) and washing with DMEM/F-12 (2 mM L-Glutamine, 1% Antibiotic-Antimycotic), the single-cell suspension from each sample was loaded onto 50% Percoll. The NSCs were separated from other cells by ultracentrifugation at 127 k rpm for 30 min at 20°C using a SW41 rotor (Beckman, CA). The fraction containing NSCs was collected and cultured with DMEM/F-12 medium containing 20 ng/ml basic fibroblast growth factor (FGF-2, PeproTech, #K1606), 20 ng/ml epidermal growth factor (EGF, PeproTech, #A2306), 1% N2 supplement (GIBCO, #17502-048), 1% Antibiotic-Antimycotic, and 2 mM L-glutamine in a 5% CO_2_ incubator at 37°C. Half of the medium was replaced every two days.

### Quantification of Mature microRNAs Using Real Time PCR

Individual reverse transcription and TaqMan® microRNA assays were performed on an Applied Biosystems 7300 Instrument as described previously [18], [19], [25]. Briefly, 15 µL Reverse transcription reactions consisted of 10 ng Total RNA isolated with TRIzol (Invitrogen, 15596-026), 5 U MultiScribe Reverse Transcriptase, 0.5 mM each dNTPs, 1X Reverse Transcription buffer, 4 U RNase Inhibitor, and nuclease free water. Reverse transcription reactions were incubated at 16°C for 30 min, 42°C for 30 min, 85°C for 5 min, and then stored at 4°C until use in TaqMan assays. 10 µL TaqMan real-time PCR reactions consisted of 1X TaqMan Universal PCR Master Mix No AmpErase UNG, 1X TaqMan miRNA assay, 1.33 µL of undiluted cDNA, and nuclease free water. Each TaqMan assay was done in either triplicate or quadruplicate for each sample tested. Relative quantities were calculated using the ΔΔCt method with RNU6B TaqMan miRNA control assay as the endogenous control and calibrated to the wildtype samples [50]. Reactions were run with the Standard 7500 default cycling protocol without the 50°C incubation stage using the SDS 7500 Fast System Software version 1.3.1, with reactions incubated at 95°C 10 min, followed by 40 cycles of 95°C 15 sec, 60°C 1 min. Fluorescence readings were collected during the 60°C step.

### Chromatin Immunoprecipitation

ChIP was performed according to published method [18], [19]. Briefly, aNSCs, grown to 80%–90% confluent (or at least 1×10^7^ cells) in 10 cm plates, fixed by adding 1% formaldehyde (Sigma-Aldrich) to culture medium for10 min at room temperature. The fixed neurospheres were collected by centrifugation. After washing with cold PBS, cells were collected with cold PBS, washed, and suspended in 1 mL cold cell lysis buffer (5 mM PIPES, pH 8.0, 85 mM KCl, 0.5% NP40), and 1X Complete Proteinase inhibitor (Roche), and incubated on ice for 5 min. Cell lysates were pelleted by centrifugation at 3000 rpm for 5 min, resuspended again in 1 mL cold cell lysis buffer 5 min on ice, and then re-pelleted to collect nuclei. Nuclei were lysed at room temperature with 500 µL nuclei lysis buffer (50 mM Tris pH 8.1, 10 mM EDTA, 1% SDS, and 1X Complete Protease inhibitor). Nuclear lysates were sonicated using a 60 sonic dismembrate sonicator (Fisher Scientific); 6 pulses, 5 seconds each at a power output of 3.0, with 1-minute incubations on ice in between each pulse. The size of the sonicated chromatin (average size ∼500–600 bp) was verified by treating 5 µL aliquots with 1 µL 20 mg/mL Proteinase A for 20 min at 50°C and running on a 1.5% agarose gel stained with SYBR Safe dye (Invitrogen). 50 µL of sonicated chromatin, pre-cleared with salmon sperm/tRNA blocked Protein A agarose for 60 min at 4°C in 950 µL IP dilution buffer (0.01% SDS, 1.1% TritonX-100, 1.2 mM EDTA, 20 mM Tris pH 8.1, 500 mM NaCl) was used in immunoprecipitation reactions. Pre-cleared chromatin was rotated at 4°C overnight with 10 µg of the appropriate antibody. Antibodies used were normal Rabbit IgG (Upstate, Cat# 12-370), rabbit Mbd1 (M-254, SC-10751), Santa Cruz Biotechnology.

Antibodies were pulled down with 60 µL blocked Protein A agarose beads for 1hr at 4°C with rotation. The beads were washed sequentially two times each in IP dilution buffer, TSE-500 solution (0.1% SDS, 1% Triton X-100, 2 mM EDTA, 20 mM Tris pH 8.1, 500 mM NaCl), freshly prepared Li/Cl wash solution (100 mM Tris pH 8.1, 300 mM LiCl, 1% NP40, 1% deoxycholic acid), and 1X TE for 10 min at 4°C. Protein-DNA complexes were eluted from the Protein A agarose beads twice with 250 µL IP elution buffer (50 mM NaHCO_3_, 1% SDS) for 15 min at 37°C with rotation. Formaldehyde induced protein-DNA crosslinking were heat reversed by incubating protein-DNA complex at 65°C overnight. DNA was purified using Phenol:Chloroform:Isoamyl alcohol (25∶24:1) isolations and precipitated with 2 volumes 100% ethanol and 10 µg linear acrylamide at −35°C overnight. Immunoprecipitated and purified DNA fragments were resuspended in nuclease free water, concentrations were determined by Nanodrop, and each sample was diluted to 1 ng/µL. 8 ng DNA was used in 20 µL SYBR Green real-time PCR reactions consisting of 1X Power SYBR Green Master Mix and 0.5 µM forward and reverse primers. Reactions were run on an Applied Biosystems SDS 7500 Fast Instrument using the Standard 7500 default cycling protocol and SDS 7500 Fast System Software version 1.3.1 without the 50°C incubation: 95°C 10 min, 40 cycles of 95°C 15 sec, 60°C 1 min. Primers sequences spaced at 1 kb intervals spanning 4 kb upstream to 2 kb downstream of mmu-mir-195 were designed using primer premier 5.0 Software and were: 4 kb upstream: FW-5′-CTGAACCCAGGTACAAAGCAG,RV-5′- CAAGAACAGAAGGGAGAGGC; 3 kb upstream: FW-5′- AGATGGGGTGTCTCTTGTTAAAG; RV-5′- CCTCTGTCTGCTTTCTCTTTGG; 2 kb upstream: FW-5′- GCACCTCATACTGAAACCAAAGC, RV-5′- CCTATATCAAGCCCTGCCAAC; 1 kb upstream: FW-5′- GCCTGTGCTGTCTTCCTCTC, RV-5′-TCCCATACCCTCGCTCTAAC; 1 kb downstream: FW-5′- TGGAACAGGAAGGAAAACGGA, RV-5′- GGAGGGTCCCCACAGAAAAC; 2 kb downstream: FW-5′- TGTGGTGACATAAAGGTAGACTG, RV-5′- GTGGTTGTGTTTGATGATAAGAGT.

Enrichment of DNA was determined by taking absolute quantity ratios of specific (MBD1) immunoprecipitations to non-specific immunoprecipitations (Normal rabbit IgG only), IP/IgG. Absolute quantification was done based upon standard curves generated from 4 10-fold dilutions ranging from 0.08–80 ng Input DNA treated in parallel with immunoprecipitated DNA during reverse crosslinking and purification steps. For Histone ChIP experiments, quantity was determined based upon Input DNA generated standard curves and reported directly for both specific and IgG nonspecific immunoprecipitations. All ChIP experiments were done from 3 independent chromatin preparations and all Real-time PCR reactions were carried out in triplicate for each sample on each amplicon.

### Proliferation and Differentiation Analyses of Cultured aNSCs

Proliferation and differentiation of aNSCs were carried out using our established method [4], [18], [24]. We used only early passage cells and comparable passage numbers of WT and KO cells. For each experiment, at least triplicate wells of cells were analyzed, and results were averaged as one data point (n = 1). At least 3 independent experiments (n = 3) were performed and used for statistical analyses for each analysis. To study cell proliferation, we dissociated neural stem cells with trypsin and plated them on poly-L-ornithin/laminin-coated glass coverslips at a density of 50,000 cells/well in proliferation medium (see above). At 20 h post-plating, 5 µM 5-bromo-2′-deoxyuridine (BrdU, Sigma-Aldrich) was added into the culture medium for 8 h. NSCs were then washed with PBS and fixed with 4% paraformaldehyde for 30 min at room temperature, followed by immunohistochemical analysis. To detect BrdU incorporation, fixed cells were pretreated with 1 M HCl for 30 min at 37°C, and then washed with borate buffer, pH 8.5, for 30 min. We then followed our standard immunohistochemistry protocol.

For the differentiation assay, at 24 h post-plating, cells were changed into differentiation medium, DMEM/F12 (1∶1), containing 5 µM forskolin (Fsk, Sigma-Aldrich, #F-6886), 1 µM retinoic acid (RA, Sigma-Aldrich, #R-2625), and sometimes with 0.5% fetal bovine serum (FBS, Sigma-Aldrich, #F-2442) for 4 days, followed by fixation with 4% paraformaldehyde for 30 min, then washing with Dulbecco’s Phosphate-Buffered Saline, pH 7.4 (DPBS) for 30 min.

Immunocytochemistry staining was carried out as described [4], [18], [23], [24], [51]. Briefly, cells were preblocked using DPBS containing 5% normal goat serum (VECTOR, #S-1000) and 0.1% Triton X-100 for 30 min, followed by overnight incubation with primary antibodies: mouse neuron-specific type ß-III tubulin (Tuj1, 1∶4000, Promega, #G712A), rabbit glial fibrillary acidic protein (GFAP, 1∶1000, DAKO, #Z-0334), or rat anti-BrdU (1∶3000, Abcam, ab-6326). After washing with DPBS, cells were incubated with secondary antibodies that included goat anti-mouse Alexa Fluor 568 (1∶500, Invitrogen, #A11031), goat anti-rabbit Alexa Fluor 647 (1∶500, Invitrogen, #A21245), or goat anti-rat Alexa Fluor 568 (1∶500, Invitrogen, #A11077), followed by counterstaining with the fluorescent nuclear dye 4′,6-dimidino-2′-phenylindole dihydrochloride (DAPI, Sigma-Aldrich, #B2261). After the cells were mounted with VECTASHIELD (VECTOR, #H-1000), the numbers of Tuj1-, GFAP-, BrdU, or activated caspase-3-positive cells were quantified using an Olympus BX51 microscope equipped with a MicroFire digital camera (Optronics) and a motorized stage using a 20X objective lens. The quantification was carried out using an unbiased stereology method with assistance from StereoInvestigator software (MBF Biosciences). The percentage of differentiated cells was calculated as the number of Tuj1- or GFAP-labeled cells divided by the total number of cells stained with DAPI. The percentage of proliferating cells was defined as the number of BrdU-labeled cells divided by total DAPI-positive cells. The percentage of dying neurons was calculated as the number of Tuj1 and Caspase-3 double labeled cells divided by the total number of Tuj1-positive cells. The data were analyzed using two-tailed unpaired t-tests.

### Electroporation, Transfection, and Luciferase Assay

Control miR-C, control, anti-C, miR-195, anti-miR-195, and anti-miR-184 were purchased from GenePharma Ltd. (Shanghai, China). NeuroD1-luciferase DNA, GFAP-luciferase and internal control E1α-Rluc DNA plasmids were described previously [18], [23], [24]. The lentivirus-MBD1 (clone 19) was published previously [4]. The pXZ-95 was used for testing MBD1 siRNA used in Liu CSC 2010 [18]. Electroporation of plasmid DNA into aNSCs and the luciferase assay were carried out using an Amaxa Nucleofector electroporator (Amaxa, #VPG-1004) based on the manufacturer’s protocol with modifications [18], [52]. Briefly, 1−2×10^6^ cells were trypsinized, resuspended in Nucleofector solution, mixed with DNA, and electroporated using a preset program for mouse NSCs (#A033). The cells were then plated onto polyornithin/laminin-coated 24-well plates in proliferation medium. After 24 h, cells were changed into differentiation medium for 24 h. Transfection of aNSCs was carried out using Fugene HD (Roche, cat# 04709713001) based on the manufacturer’s protocol with modification. Briefly, aNSCs were plated into 24-well P/L-coated plate for 24 hours. Then 1ug DNA was added into 50 ul DMEM/F12 medium without PSF. And then 3 ul transfection reagent Fugene HD was added into the same tube, pipetted 3–5, incubated for 30 minute, and then added onto the cells. Sixteen hours later, the transfected cells were changed into differentiation medium for 24 hours. The cells were then collected and luciferase activity was detected using the Dual-Luciferase Reporter 1000 System (Promega, Cat# E1980) based on the manufacturer’s protocol. Briefly, collected cells were lysed in 100 µl of 1X passive lysis buffer at room temperature for 15 min. Then 20 µL of the lysate was added to 100 µl of Luciferase Assay Buffer II and mixed briefly. Firefly luciferase (F-luc) activity was immediately read using a SpectraMax M2E plate reader (Molecular Devices Corp.). Next, 100 µl of Stop & Glo Buffer with Stop & Glo substrate was added and mixed briefly. Renilla luciferase (R-luc) activity was immediately read. F-luc activity was normalized to R-luc activity to account for variation in transfection efficiencies. Each experiment was independently repeated 3 times. For each electroporation, 3 µg (*NeuroD1*- or GFAP-) luciferase DNA, 5 µg *Mbd1 expression or Mbd1 shRNA vector* DNA, 0.2 µg R-Luc, or control expression plasmids were used. The luciferase counts were then normalized to R-luc counts to obtain final NeuroD1 or GFAP promoter activities.

### Lentivirus Expressing shmiR-195 or miR-195 Sponge

PCR based generation of the shmiR-195 driven by a U6 Pol III promoter was done using a PCR Shagging approach as previously described with the following PAGE purified long oligos:

shRNA miR-195 (U6-shmiR-195): 5′-GACGTAATCGATAAAAAAATAGCAGCACAGAAATATTGGCTCTCTTGAAGCCAATATTTCTGTGCTGCTA AAACAAGGCTTTTCTCCAAGGGA-3′ shRNA Control (U6-NC, published [53]): 5′-TATCGATAAAAAAAAATTCTCCGAACGTGTCACGTTCTCTTGAAACGTGACACGTTCGGAGAATTAAACAAGGCTTTTCTCCAAGGGA-3′.

Long oligos were used as reverse primers in combination with a common forward primer complementary to the 5′ end of the U6 promoter (5′-AAAGTTAACTAGTGGATCCGACGCCGCCATCTC-3′) to amplify the entire U6 promoter and shRNA in a single PCR product. Amplification was done using 20 ng of a previously generated U6-shRNA lentiviral construct [52] with TaKaRa Ex Taq™ 1X PCR Buffer, 2 mM MgCl_2_, 0.2 mM dNTP mix, 0.2 mM forward primer, 0.2 mM reverse primer, 2.5 U Ex Taq™, 95°C 10 min, 40 cycles of 94°C 1 min, 60°C 1 min, 72°C 1 min, followed by 72°C 10 min and then stored at 4°C. 2 µL of PCR product was used in a TOPO TA cloning reaction with pCR2.1 vector and chemical transformation of TOP-10 competent cells (Invitrogen, K4500-01SC). U6-shRNA expression constructs were removed from the TOPO vector for transfer to a lentiviral vector or a retroviral vector (see below) by HpaI and ClaI restriction digestion. The lentiviral vectors expressing miR-195 or control shRNA were then verified by sequencing.

Lentivirus production was performed as described previously (Barkho et al. 2006) [18], [52] Briefly, lentiviral transfer vector DNA and packaging plasmid DNA were transfected into cultured 293T cells using calcium phosphate methods. The medium containing lentivirus was collected at 40, 64, and 88 hours post-transfection, pooled, filtered through a 0.2-µm filter, and concentrated using an ultracentrifuge at 19 k rpm for 2 hours at 20°C using a SW27 rotor (Beckman). The virus was washed once and then resuspended in 500 µl phosphate buffered saline. We routinely obtained 0.5–1×10^9^ infectious viral particles/ml. To study the effects of miR-195 on the proliferation and differentiation of NSCs, ∼60 µl Lentivirus was added to the wild-type NSCs cultured in proliferating condition on a 10 cm tissue culture plate. After a 3-day incubation, infected NSCs were either collected for RNA analysis or trypsinized and plated into either chamber slides (Nulge Nunc, #154526), at a density of 5–7×10^4^ cells/well, for differentiation or proliferation analysis.

### Construction of Retroviral Vector Expressing shmiR-195 and miR-195-sponge and in vivo Retroviral Grafting

Retroviral vector expressing shmiR-195 and GFP was cloned as above for lentivector expressing shmiR-195 and as described in our publications [18], [19], [29]. miR-195-sponge was designed using a bulge design method based on a published paper [30]. Binding sites for miRNA-195 were complementary in the seed region with a bulge at positions 9–12 to prevent RNA interference–type cleavage and degradation of the sponge RNA. We constructed miR-195-sponge into CAG-mRFP vector [26] by inserting 6 tandem arrayed miR-195 binding sites into the Not1 site located in the immediate 3′-UTR of RFP. The vectors expressing miR-195 sponge were then verified by sequencing. CAG-RFP empty vector was used as a control.

Retrovirus production was performed as described previously [18], [19], [25], [51]. Briefly, Retroviral transfer vector DNA and packaging plasmid DNA was co-transfected with packaging plasmids pCMV-gag-pol and pCMV-Vsvg into HEK293T cells using the calcium phosphate method. The medium containing retrovirus was collected at 40, 64, and 88 hours post-transfection, pooled, filtered through a 0.2-µm filter, and concentrated using an ultracentrifugation at 19.4 krpm for 2 hours at 20°C (Beckman SW27 rotor). The virus was washed once with phosphate buffered saline (PBS) and then resuspended in 150 µl PBS.

In vivo retroviral grating was performed based on published method with modification [18], [19], [25], [29], [51]. Briefly, 7 to 8-week-old C57B/L6 male mice were anesthetized with isofluorane and virus (1.5 µl with titer greater than 5×10^5^/µl) was injected stereotaxically into the DG using the following coordinates relative to bregma: anteroposterior, − (1/2)×d mm; lateral, +/−1.8 mm (if d>1.6) or +/−1.7 mm; ventral, −1.9 mm (from dura). For each mouse, the virus was injected into both the left and the right DG. At 24 hours post-viral injection, mice received 4 BrdU injections (50 mg/kg, i.p.) within 12 hours. For proliferation analysis, mice were sacrificed at 12 hours after the last BrdU injection (48 hours after viral grafting). For differentiation, mice were sacrificed at 7 days after the first BrdU injection (8 days after viral grafting). Mice were deeply anesthetized with Avertin and perfused with saline followed by 4% PFA. Brains were dissected out, post-fixed overnight in 4% PFA, and then equilibrated in 30% sucrose. Forty-micrometer brain sections were generated using a sliding microtone and were stored in −20°C freezer as floating sections in 96-well plates filled with cryoprotectant solution (glycerol, ethylene glycol and 0.2 M phosphate buffer, pH 7.4, 1∶1:2 by volume).

Immunohistochemistry and confocal imaging analysis were carried out as described [18], [19], [25], [51]. Floating brain sections containing eGFP+ or RFP+ cells were selected for staining and matched by DG region. Sections were pretreated with 1 M HCl, as described in previous study. The primary antibodies used were chicken anti-GFP (Invitrogen, #A10262), rabbit anti-dsRed (Clontech, #632496), rat anti-BrdU (Abcam, ab-6326), and rabbit anti-Doublecortin (DCX, cell signaling, #4604). The secondary antibodies used were anti-chicken Alexa Fluor 488 (Invitrogen, #A11039), goat anti-rat Alexa Fluor 647 (Invitrogen, #A21242), and goat anti-rabbit Alexa Fluor 568 (Invitrogen, #A11036). The z-stacks images of GFP/DCX and RFP/BrdU staining were taken at 1 µm using a Zeiss ApoTome confocal microscope with an oil immersion objective (40×; NA = 1.3; Zeiss). For co-localization analysis, roughly 50–80 FP+ (GFP+ or RFP+) cells per animal were imaged and analyzed. The proportion of FP+DCX+ among total FP+ cells and the proportion of FP+BrdU+ cells among total FP+ cells were counted. The data was analyzed using student t-test.

### 3′-UTR Dual Luciferase Assays of Candidate miR-195 Target mRNAs

Cloning the 3′UTR into luciferase vector was performed as described in our publications [18], [19]. Briefly, 3′-UTR sequences of candidate mRNAs were PCR amplified directly from proliferating cDNA generated from aNSC total RNA using oligo-dT SuperScript III reverse transcription according to manufacturer’s protocol (Invitrogen, Cat. #1808-093). The sequences of primers for *Mbd1* are: Forward sequence: CCAGCTCGAG GAAAGAAGAAGTTTTGTAGGAG; Reverse sequence: CCAG GCG GCC GC CAAAGAATTTTCAGGATCAACC.The primers were designed incorporating XhoI and NotI restriction sites and 4 bp extra random sequence for aiding in restriction digest. XhoI and NotI digested PCR products were cloned into XhoI and NotI digested psiCHECK-2 dual luciferase vector (Promega, Cat# C8021). The constructs were co-transfected with small RNAs into aNSCs using FuGene HD transfection reagent (Roche, cat# 04709705001). Luciferase expression was detected using the Dual luciferase reporter 1000 system (Promega, Cat# E1980) per manufacturer’s protocol. Briefly, 48 hours after transfection cell culture medium was removed and cells were lysed with 20 µl of 1X passive lysis buffer at room temperature for 15 min. 100 µL Luciferase Assay Buffer II was added and mixed briefly. Firefly luciferase (F-luc) activity was immediately read using a Spectramax M2E (Molecular Devices Corp). 100 µL Stop & Glo Buffer with Stop & Glo substrate was then added and mixed briefly. Renilla luciferase (R-luc) activity was immediately read. R-luc activity was normalized to F-luc activity to account for variation in transfection efficiencies, and miR-195 mediated knockdown of R-luc activity was calculated as the ratio of normalized R-luc activity in the miR-195 or anti-miR-195 transfected to normalized R-luc activity in the control miR treated conditions. All luciferase readings were taken from either 3 or 4 individual wells for each psiCHECK-2-3′-UTR construct and control construct tested.

The miR-195 target site in the Mbd1-3′UTR was changed using the QuickChange lighting Site-Directed Mutagenesis Kit (Statagene, Cat. #210518) to change seven bases (CGACGAU) from the miR-195 seed site in the Mbd1- 3′UTR luciferase reporter. Deletion of the target site was verified by sequencing. The primers used for the deletion are the following:

Forward: TTAGACAACTGCCCCATACTCACATACGATCTACCCTCTTCCTGTG

Reverse: CACAGGAAGAGGGTAGATCGTATGTGAGTATGGGGCAGTTGTCTAA

### Western Blotting Analyses

Protein samples were separated on SDS-PAGE gels and then transferred to PVDF membranes (Millipore). Membranes were processed following the ECL Western blotting protocol (GE Healthcare). Anti-Mbd1 (M-254, SC-10751, Santa Cruz Biotechnology) was used as primary antibodies at the concentrations recommended by the manufacturers. HRP-labeled secondary antibodies were obtained from Sigma. For loading controls, membranes were stripped and reprobed with the antibody against β-Actin.

### Real-time PCR and Primer Sequences

The first-strand cDNA was generated by reverse transcription with oligo dT primer (Roche).To quantify the mRNA levels with the real-time PCR, aliquots of first-stranded cDNA were amplified with gene-specific primers and Power SYBR Green PCR Master Mix (Invitrogen) using a 7300 Equipment (Applied Biosystems). The PCR reactions contained 20–40 ng of cDNA, Universal Master Mix (Invitrogen), and 200 nM of forward and reverse primers in a final reaction volume of 20 µl. The ratio of different samples was calculated by the data analysis software built in with the 7300 Fast Real-Time PCR System. The sequences of primer used are as the following:

*Mbd1:* forward: CGTCTCAGCGTCACTCCCAAGC; reverse: ACGCAATCCTGCTCCCTCCC

*NeuroD1*: forward: TTAAATTAAGGCGCATGAAGGCC; reverse: GGACTGGTAGGAGTAGGGATG

*Tuj1:* forward: TATGAAGATGATGACGAGGAATCG; reverse: TACAGAGGTGGCTAAAATGGGG

*GFAP:* forward: CCAAGCCAAACACGAAGCTAA; reverse: CATTTGCCGCTCTAGGGACTC

### Statistical Analysis

Statistical analysis was performed using ANOVA and Student t-test, unless specified with the aid of SPSS v.17. All percentages were arcsine transformed before statistical analysis. The Bonferroni correction was used to control type I error [54]. We normalized the treatment group by the control group for luciferase, RT-PCR, and cell counting analyses, and then one-sample t-test against mean of 1 was applied on the normalized values. All data were shown as mean with standard error of mean (mean ± SEM). Probabilities of P<0.05 were considered as significant.

### Ethics Statement

Animal procedures were approved by the Institutional Animal Care and Use Committee of the University of New Mexico and University of Wisconsin-Madison and conformed to National Institutes of Health guidelines.

## Supporting Information

File S1 Seven Supplemental Figures, one supplemental table and legends.
(PDF)
