# Supplementary material for: An Epigenetic Feedback Regulatory Loop Involving MicroRNA-195 and MBD1 Governs Neural Stem Cell Differentiation
Source: PLoS One. 2013 Jan 17;8(1):e51436. doi: 10.1371/journal.pone.0051436 (PMC3547917; doi:10.1371/journal.pone.0051436)
Supplement: File S1 — Seven Supplemental Figures, one supplemental table and legends. (PDF) [file pone.0051436.s001.pdf]

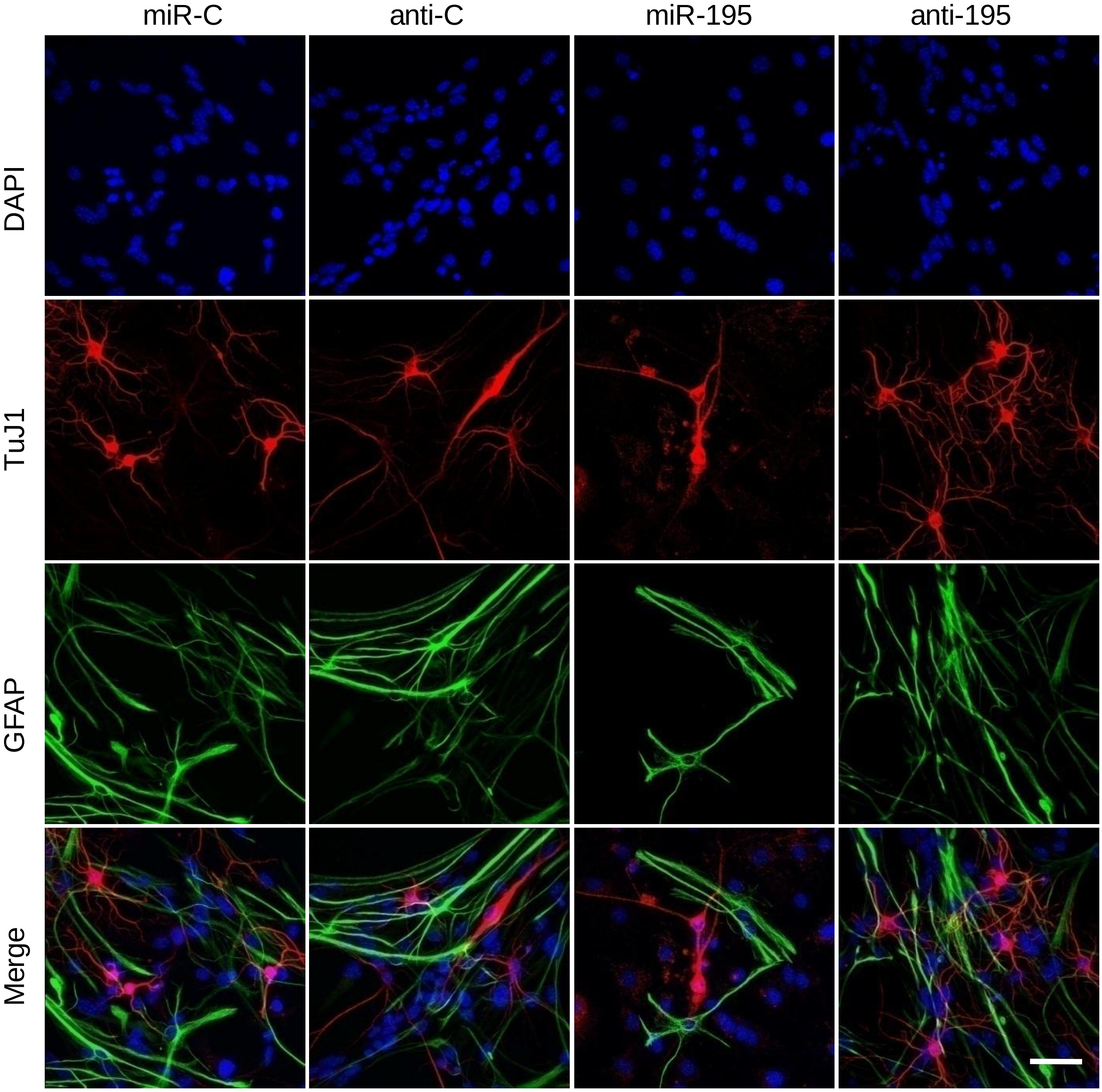

Figure S1. High levels of miR-195 inhibits aNSC differentiation.
Representative images of DG-aNSCs transfected with either miR-195 or an inhibitor for miR-195 (anti-195) that have undergone differentiation. miR-195-transfected aNSCs had reduced GFAP+ astrocytes and TuJ1+ neurons, compared to control miR-C-treated cells. Anti-195-transfected NSCs displayed increased GFAP+ astrocytes and TuJ1+ neurons relative to control anti-C-treated cells. Neuronal lineage marker Tuj1 (red) and astrocyte lineage marker GFAP (green) were used to assess differentiation. DAPI, blue. (Scale bar = 50 μm).

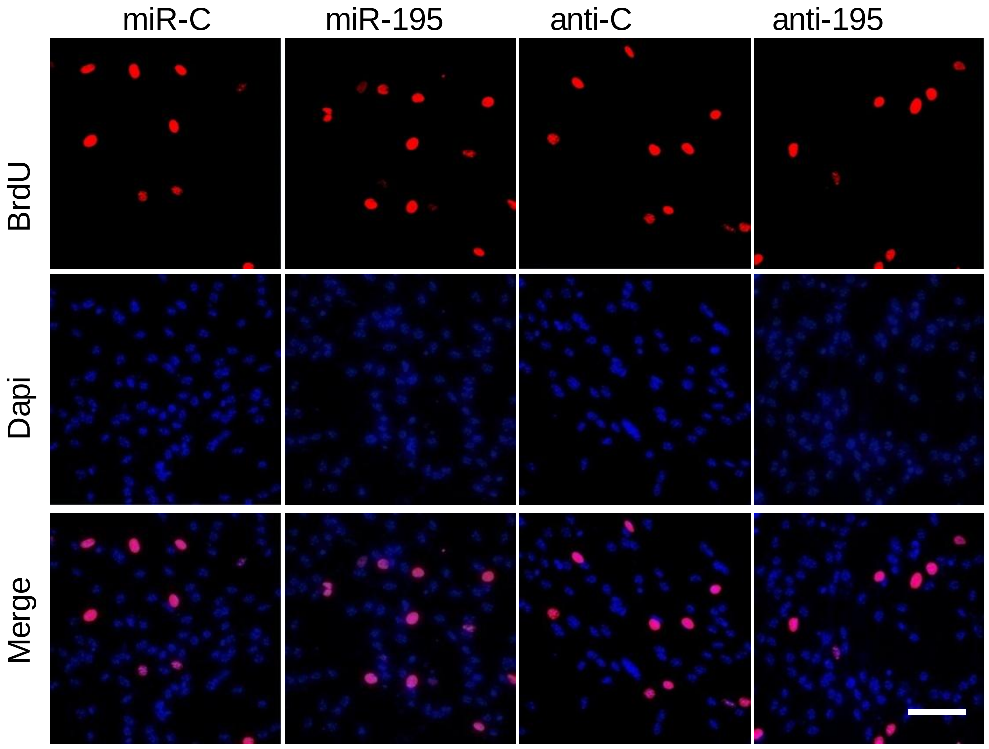

Figure S2. High levels of miR-195 promotes aNSC proliferation. Representative images of DG-aNSCs transfected with either miR-195 or an inhibitor for miR-195 (anti-195) that have been subjected to BrdU pulse-labeling for proliferation analysis. miR-195-transfected aNSCs had increased BrdU labeling, compared to control miR-C-treated cells. Anti-195-transfected NSCs displayed decreased BrdU labeling relative to control anti-C-treated cells. BrdU, red. DAPI, blue. (Scale bar = 50 μm).

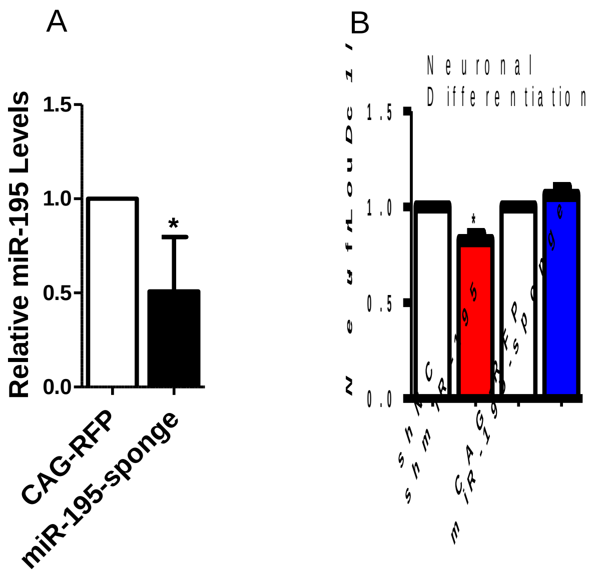

Figure S3. Assessment of viral expressed miR-195 and miR-195 sponge.
(A). DG-aNSCs infected with retrovirus expressing miR-195-sponge exhibited reduced mature miR-195 levels compared to control CAG-RFP virus-infected cells ($n=5$, $p<0.05$).
(B) Overexpression of retrovirus-shmiR-195 (used in Fig.3) in WT aNSCs resulted in reduced aNSC neuronal differentiation ($n=4$, $p<0.05$) and overexpression of retrovirus-miR-195-sponge (used in Fig. 3) led to slight increase ($n=4$, $p=0.18$) in aNSC neuronal differentiation as assessed by *NeuroD1* promoter activities

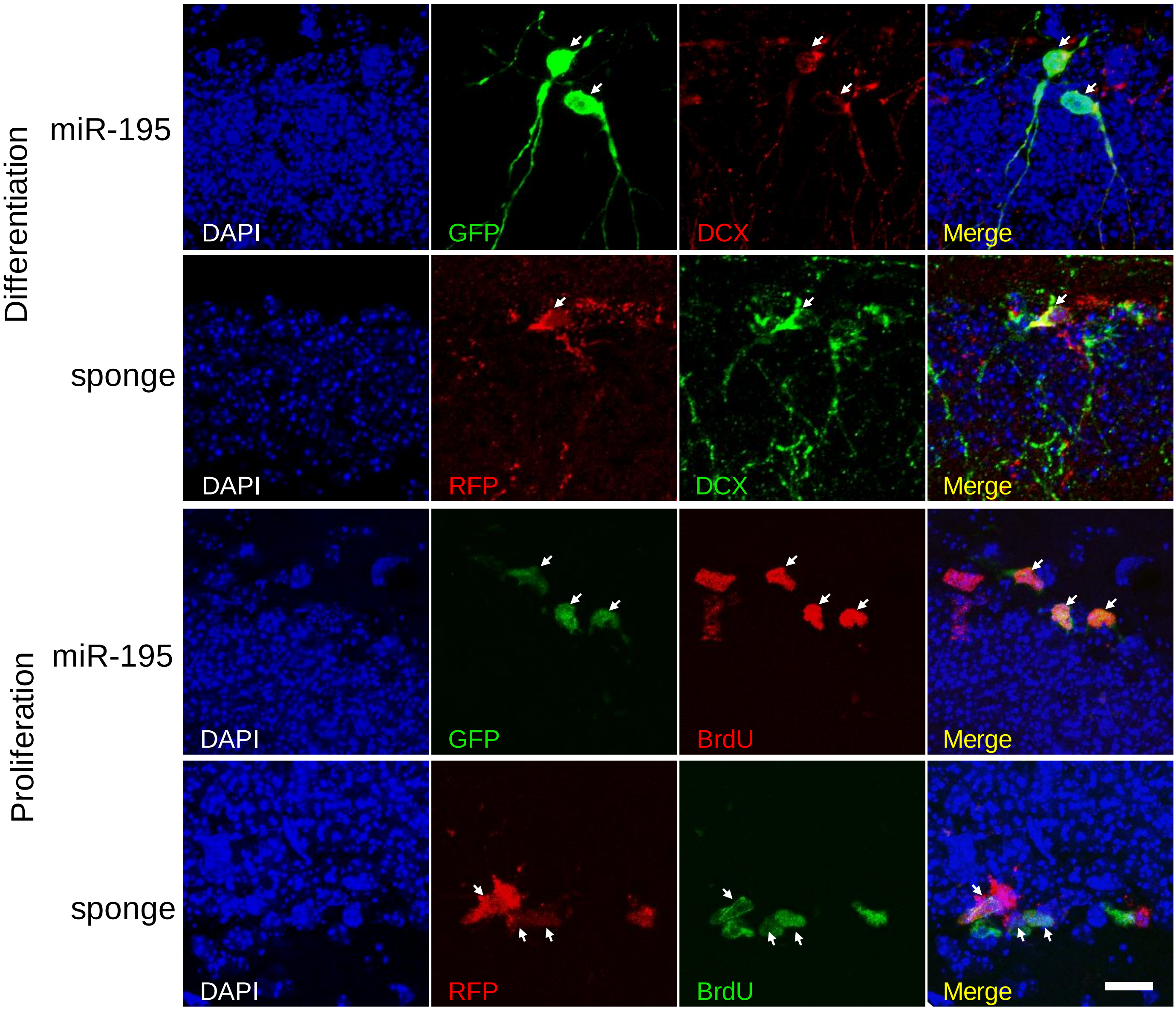

Figure S4. miR-195 modulates the differentiation of adult NSCs *in vivo*
Representative images of retrovirus-infected cells analyzed by cell proliferation and neuronal differentiation markers at 1 week post-viral grafting. (Scale bar = 10 μm).

Sh-miR-195 retrovirus-infected cells also expressed GFP (green). DCX or BrdU, red. DAPI, blue.

miR-195-sponage virus-infected cells also expressed RFP (red). DCX or BrdU, green, DAPI, blue.

Arrows indicates double labeled cells.

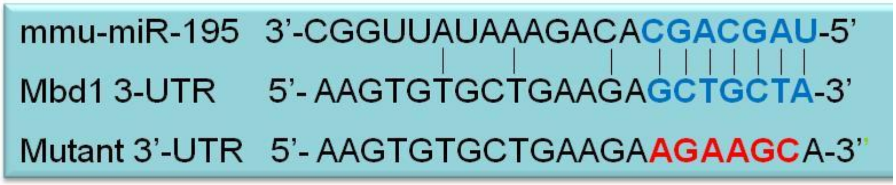

Figure S5. A miR-195 target site was found in the *Mbd1* 3' untranslated region (3' UTR) as predicted by TargetScan software. To determine whether miR-195 repressed MBD1 translation through this target site, a mutant *Mbd1* 3'UTR construct was created by mutating the seed sequence (nucleotides in blue) within the *Mbd1* 3'UTR (mutant nucleotides are in red).

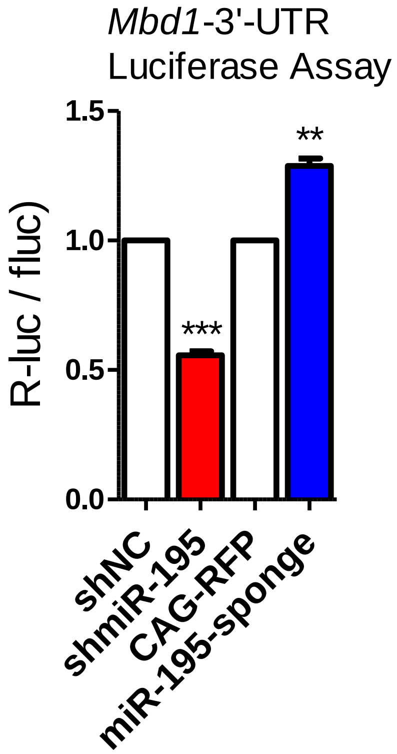

Figure S6 *Mbd1*-3'-UTR-dependent expression of Renilla luciferase reporter gene (R-Luc) was suppressed by retrovirus-shmiR-195 and enhanced by the retrovirus-miR-195-sponge in aNSCs (n=4,p<0.01).

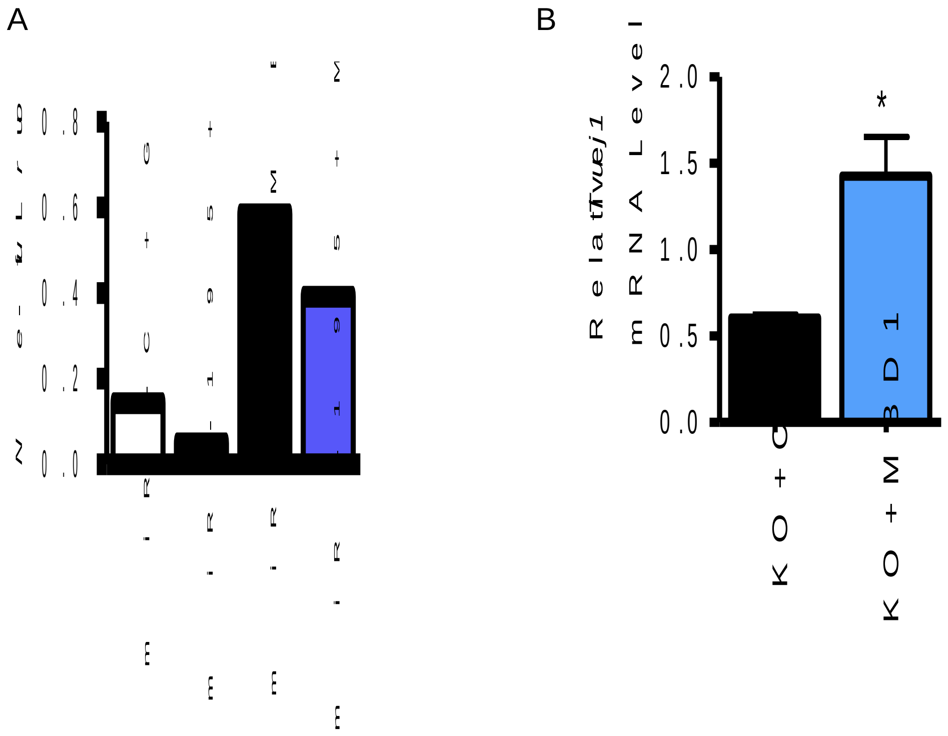

Figure S7. MBD1 rescues neuronal differentiation deficits resulted from either miR-195 overexpression or MBD1 deficiency. (A). MBD1 rescues miR-195 overexpression-induced neuronal differentiation deficits (data is an average of one experiment with 3 replicates). (B) Exogenous MBD1 could rescue neuronal differentiation deficits exhibited by *Mbd1 KO* aNSCs as assessed by *Tuj1* mRNA expression in differentiating aNSCs (D, n = 4).rescues neuronal differentiation deficits of MBD1 KO NSCs (n=4-6). Data are presented as mean ± SEM; statistics were done using one-sample t-test*, $p < 0.05$, **, $p < 0.01$.

Table S1. Predicted targets of miR-195 by TargetScan and miRanda program

MBD1	↓	↓
EphA7	↓	NA
Wee1	↓	NA
Mib1	↓	NA
BDNF	↓	NA
CyclinD1	NA	No change
Sox5	NA	NA
NOVA1	NA	NA
FoxJ1	NA	NA
Wnt3a	NA	NA
Nrg1	NA	NA
Reln	NA	NA

Note: down arrow indicates that miR-195 repressed the luciferase activity through the 3'UTR of indicated target mRNA. No Change, transfected miR-195 did not affect the expression of this protein. NA, Not analyzed.
